# Photodegradation of Selected PCBs in the Presence of Nano-TiO_2_ as Catalyst and H_2_O_2_ as an Oxidant

**DOI:** 10.3390/ijerph7113987

**Published:** 2010-11-15

**Authors:** Samuel S. R. Dasary, Julia Saloni, Amanda Fletcher, Yerramilli Anjaneyulu, Hongtao Yu

**Affiliations:** Department of Chemistry and Biochemistry, Jackson State University, 1400 J. R. Lynch Street, P.O. Box 17910, Jackson, MS 39217, USA; E-Mails: samy_12345@yahoo.com (S.S.R.D.); saloni@icnanotox.org (J.S.); amandawfletcher@yahoo.com (A.F.); yerramilli.anjaneyulu@jsums.edu (Y.A.)

**Keywords:** photodegradation, PCBs, H_2_O_2_, dechlorination, TiO_2_

## Abstract

Photodegradation of five strategically selected PCBs was carried out in acetonitrile/water 80:20. Quantum chemical calculations reveal that PCBs without any chlorine on *ortho*-positions are closer to be planar, while PCBs with at least one chlorine atoms at the *ortho*-positions causes the two benzene rings to be nearly perpendicular. Light-induced degradation of planar PCBs is much slower than the perpendicular ones. The use of nano-TiO_2_ speeds up the degradation of the planar PCBs, but slows down the degradation of the non-planar ones. The use of H_2_O_2_ speeds up the degradation of planar PCBs greatly (by >20 times), but has little effect on non-planar ones except 2,3,5,6-TCB. The relative photodegradation rate is: 2,2′,4,4′-TCB > 2,3,5,6-TCB > 2,6-DCB ≈ 3,3′,4,4′-TCB > 3,4′,5-TCB. The use of H_2_O_2_ in combination with sunlight irradiation could be an efficient and “green” technology for PCB remediation.

## 1. Introduction

Polychrlorinatedbiphenyls (PCBs) are toxic xenobiotics which were once widely used as heat transfer fluids, hydraulic fluids, solvent extenders, plasticizers, flame-retardants, and dielectric fluids [1–4]. Due to their chemical stability and hazardous nature, they are categorized as persistent organic pollutants. PCB chronic toxicity includes damages to the heart, liver, kidney, central nervous, and reproduction systems [2,4–8]. Although there are some reports on industrial and domestic waste treatment of PCBs, there is still a need for more effective and “green” remediation [9–11]. Advanced oxidation processes are some of the popular treatment methodologies for environmental pollutants and they have been widely investigated recently [12–17]. These methods utilize reactive oxygen species to destroy toxic organic substrates by transforming them into desired inorganic minerals [18].

Sunlight-induced degradation of PCBs is the cheapest and cleanest method. This process is dependent on many factors including structure of PCBs, catalysts, nature of the solvent, and presence of oxidants [19]. Several studies reported photolysis of PCBs in various solvents and media [20–23]. Due to low solubility of PCBs in water, most degradation studies have been carried out in organic solvents such as hexane [24–27] or alcohols [28,29]. Photochemistry of non-ortho substituted PCBs under 254 nm UV irradiation were also studied in alkaline 2-propanol [30]. Photodegradation in hexane showed slower reaction for symmetrical and coplanar PCBs and reactivities of the chlorine atoms at various positions of PCB rings are generally in the order: ortho- > meta- > para [24,25]. Decomposition of highly chlorinated PCBs in octadecylsilylated silica particles by light irradiation showed a tendency to lose chlorine from meta- or para-positions [31]. 2,2′,4,4′-Tetrachlorobiphenyl degradation in cyclodextrin resulted in 2,4,4′-trichlorobiphenyl and chloride, indicating dechlorination at the ortho position [32]. Lin *et al.* [33] determined the photodegradation rates of PCBs by simulated sunlight in the presence of diethylamine. GC-MS analysis of aqueous and organic phases of PCBs in different fossil fuels showed a higher degree of photodegradation in the water-soluble fraction than the organic fractions [34].

TiO_2_ photocatalysis is a widely used remedial method for many organic pollutants [35–41] including PCBs [20,42,43]. However, TiO_2_ photocatalysis is limited by significant radiation loss due to electron-hole recombination process [13,44,45]. One way to overcome this problem is the use of an efficient electron acceptor to capture electrons and inhibit the recombination effect. Several studies have investigated the role of H_2_O_2_ as an alternative electron acceptor [42,46–50]. H_2_O_2_ is often used as an oxidant in TiO_2_ photocatalysis to enhance the reaction rate by generating more hydroxyl radicals.

Most PCBs degrade slowly with long half-lives by light irradiation. It is also noticeable that very low concentrations of PCBs were used for those studies due to poor solubility in aqueous solutions. In view of the existing knowledge gap, the present work aims at evaluating the simulated solar light-induced degradation of five strategically selected PCBs (Table 1) in acetonitrile/water (80:20) in the presence of TiO_2_ at pH 7, pH 9, and in the presence of H_2_O_2_ or TiO_2_ + H_2_O_2_. Two of the five PCBs have no chlorine substitution at *ortho*-positions and two of them are symmetrical. PCBs degradation was monitored using HPLC and degradations kinetics were determined based on the peak heights. The influence of structure, chlorine substitutions and planarity on PCBs photodegradation rates in the presence of nano-TiO_2_ and hydrogen peroxide was discussed.

## 2. Materials and Methods

### 2.1. Materials

All PCBs (99% purity) were purchased from Fischer Scientific and used without further purification. All organic solvents were HPLC grade from Sigma-Aldrich. Non-porous P-25 TiO_2_ powder (80% anatase, average diameter 200 nm and surface area ~50 m^2^/g) was a gift from Degussa Corporation (Akron, Ohio, USA). H_2_O_2_ (30%, reagent grade) was from Fischer Scientific.

### 2.2. Quantum Chemical Calculations

PCBs structures were optimized using density functional theory (DFT) with B3LYP functional and the second-order Möller-Plesset perturbation approach. No symmetry constrains were imposed during geometry optimization. Geometry searches for a variety of possible configurations were performed to obtain the global minimum. DFT and MP2 vibrational frequency calculations were carried out to verify the true minimum.

### 2.3. Photolysis of PCBs

PCBs were dissolved in acetonitrile/water (80:20) and stored at 4 °C. The PCB solution was poured into a flat-bottomed quartz cuvette (1 cm light path) with a stopper to prevent solvent evaporation. Control samples were prepared using the same conditions as the test samples but covered with aluminum foils and kept in dark. These samples were analyzed with HPLC to validate the photo aided degradation. All experiments with TiO_2_ were kept in the dark for 180 min to establish equilibrium between adsorption and desorption. The amount of PCBs adsorbed by TiO_2_ at equilibrium was measured in suspensions containing 3 mg of TiO_2_ in 3 mL of acetonitrile solution containing 100 μM of the PCB. After 180 min in the dark, the suspensions were centrifuged and the residual PCB concentrations in the supernatant were determined by HPLC. The samples after dark absorption were exposed to simulated sunlight in a reactor with a 1,000 W xenon lamp (Suntest CPS+ lamp system, Atlas MTT BV, Germany). The photoreactor lamp emits light in the range of 300–800 nm with an irradiance of 250–765 W/m^2^ and the temperature was maintained at 35 °C. Samples were collected at regular intervals of irradiation. After irradiation, the samples containing TiO_2_ were centrifuged at 9000 RPM for 10 min and the supernatant was filtered through 0.45 μm Millipore filter discs before HPLC analysis. The pH of the solutions was adjusted with 0.01 M NaOH solution. Photodegradation experiments carried out were: (1) At pH 7 and 9; (2) In the presence of TiO_2_ at pH 7 and 9; (3) In the presence of H_2_O_2_ alone; (4) In the presence of both TiO_2_ and H_2_O_2_. The amount of TiO_2_ was 1 mg/mL and the final concentration for H_2_O_2_ was 12 mM.

### 2.4. HPLC Analysis

The concentration of PCBs was assessed using HPLC (Shimadzu Corp., Kyoto, Japan). The samples were filtered with 0.25-μm Teflon syringe filter (Millipore) prior to analysis and eluted with a 4.6 × 150 mm reverse phase C_18_ (Zorbax XDB-C_18_) column using acetonitrile/water (80:20) as the mobile phase at a flow rate of 1 mL/min. PCBs were detected by absorbance at 250 nm. Replicates gave an error of ±5%. In the case of direct photolysis, samples for 0 min irradiation were collected just before the irradiation, whereas 0 min samples for TiO_2_ photocatalysis and peroxide catalysis were collected before and after addition of the catalyst to study the effect of the catalyst on degradation. The peak height (mAu) at 0 min is considered as maximum concentration (100%) of the PCB. The change in peak height with time was plotted to show the degradation of PCBs. Complete destruction of a PCB was considered when the parent peak disappeared. For all samples, triplicates were run and peak height was presented as an average of these values.

### 2.5. Degradation Kinetics

Photodegradation rate constant was calculated for pseudo-first order reactions based on the plot of **ln (*A0/At*) =** ***k*t.** A plot of ln (*A*_0_/*A_t_*) *versus* irradiation time (*t*) was fitted to a straight line whose slope equals to the first-order rate constant *k* (min^−1^). Half-life (t_1_*_/_*_2_, min) values were calculated by k/0.693.

## 3. Results and Discussions

### 3.1. Planarity of Selected PCBs Based on Quantum Chemical Calculations

All five PCBs were structurally optimized to obtain the lowest energy conformation with the computed global minimal energy structure shown in Table 1. The dihedral angle between the two benzene rings for each PCB was determined for the optimized structure. As expected, PCBs without chlorine on either of the *ortho*-positions, 3,3′,4,4′-TCB and 3,4′,5-TCB, are near planar with the dihedral angles between the two benzene rings being about 37°. For 2,2′,4,4′-TCB, which has one chlorine each on each of the benzene ring’s *ortho*-positions, the dihedral angle is 68°. If both the *ortho*-positions of the same benzene ring are occupied by chlorines, like in 2,6-DCB and 2,3,5,6-TCB, the two benzene rings are nearly perpendicular to each other with dihedral angles of 77° and 90°, respectively.

### 3.2. PCB Photodegradation Monitored by HPLC

**Figure 1:**
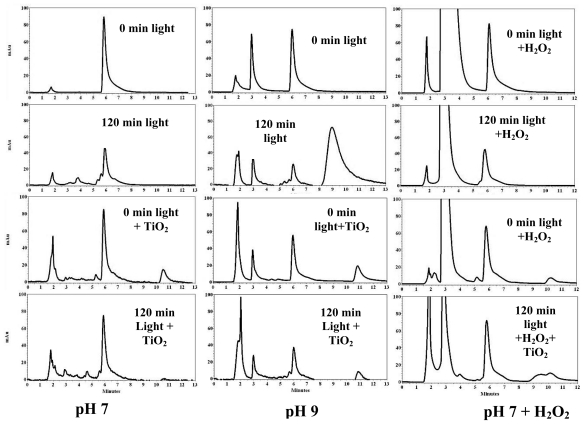
HPLC chromatograms of 2,6-dichlorobiphenyl under different conditions.

Photodegradation of PCBs was monitored using HPLC. From the chromatogram for 2,6-DCB in Figure 1, one can see that 2,6-DCB appears at an elution time of 6.0 ± 0.2 min. At pH 7, 120 min irradiation yielded intermediates between 4–6 min (Figure 1, left), but in the presence of TiO_2_, more complex products were detected with elution time of 4–6 min and 10–11 min. The peaks at 10.5 and 11.0 min are probably high molecular weight compounds that degrade on further irradiation. At alkaline pH 9 (Figure 1, middle), a major peak appeared between 8.0–10.5 min which we assume to be a higher molecular weight compound and seemed to be different from the ones under pH 7. In the presence of H_2_O_2_ alone (Figure 1, right), no significant reduction in the parent peak was observed. Further, addition of TiO_2_ slowed down the degradation and the peak at 10.5 min was split to two peaks after 120 min irradiation. This might be attributed to the dechlorination of the PCBs resulting in the formation of lower biphenyls.

**Figure 2:**
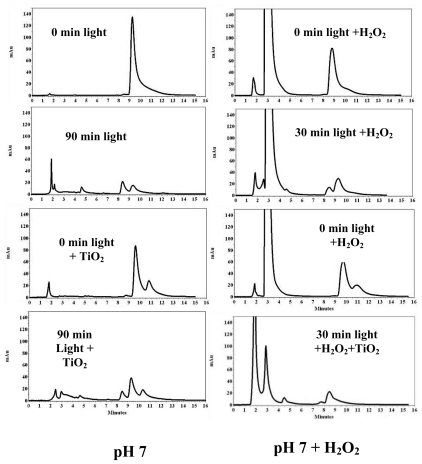
HPLC chromatograms of 2,2′,4,4′-tetrachlorobiphenyl (9.4 ± 0.2 min) irradiated in the presence of TiO_2_ and/or H_2_O_2_ at pH 7.

HPLC analysis of 2,2′,4,4′-TCB (Figure 2) showed the initial compound peak at 9.4 ± 0.2 min. At pH 7 (Figure 2, left), photolysis resulted in probably lower biphenyls at 4.0, 5.5 and 8.0 min. Similar photoproducts were observed in the case of light + H_2_O_2_ but with much faster degradation rate, indicating that peroxide speeds up the degradation. Addition of TiO_2_ as a catalyst with or without peroxide might have transformed the parent compound into a higher molecular weight product (Figure 2, right). A peak at 10.3 min elution time was seen in both cases along with the other peaks at 4.5 min, 8.0 min and 8.7 min. Degradation with H_2_O_2_ went to near completion after 30 min of irradiation.

**Figure 3:**
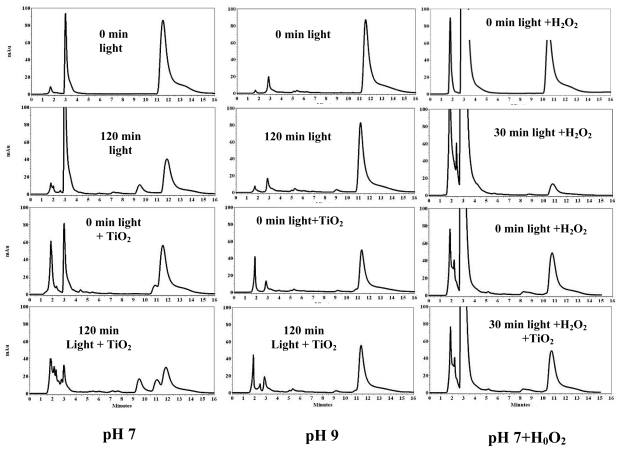
HPLC chromatogram of 3,3′,4,4′-tetrachlorobiphenyl (11.5 ± 0.2 min) irradiated at pH 7 or 9 and in the presence of H_2_O_2_ and/or TiO_2_.

HPLC chromatogram f 3,3′4,4′-TCB has a peak at 11.3 ± 0.2 min. At pH 7, light irradiation for 240 min produced peaks at 6.0, 7.0, 7.5 and 9.5 min (Figure 3, left). At pH 9, the parent compound was more efficiently degraded, but no photoproducts were detected (Figure 3, middle). There was no evidence for the formation of intermediates when H_2_O_2_ was used (Figure 3, right).

**Figure 4:**
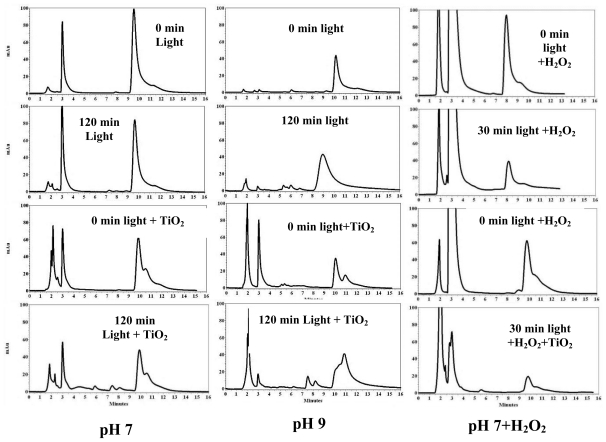
HPLC chromatogram of 3,4′,5-trichlorobiphenyl (9.8 ± 0.2 min) irradiated at pH 7 or 9 in the presence or absence of H_2_O_2._

3,4′,5-TCB has an initial peak at 9.8 ± 0.2 min. Irradiation at pH 7 (Figure 4, left) showed no degradation intermediates, but addition of TiO_2_ resulted in a shoulder on the original peak, and light irradiation produced 4 degradation products at 4.5, 5.8, 7.4 and 8.0 min. At pH 9.0, a major peak at 7.0 min was observed; whereas addition of TiO_2_ resulted in the formation of a peak around 11.0 min which may be a higher molecular weight compound (Figure 4, middle). Peroxide accelerates the degradation without formation of intermediates (Figure 4, right).

From the chromatograms of 2,6-DCB, 2,2′,4,4′-TCB and 3,3′,4,4′-TCB we observed that light-irradiation resulted in peaks eluting at lower elution times indicating a possible dechlorination of the parent PCBs, but addition of TiO_2_ catalyst generated peaks at lower and higher elution times hinting a possible generation of both dechlorinated biphenyls and higher molecular weight compounds. We have also observed the peaks with higher elution time disappeared indicating a possible degradation of the higher molecular weight compounds upon further irradiation. As an example, in Figure 2 (left) the peaks below 10 min are probably the dechlorinated products of the parent 2,2′,4,4′-TCB, whereas the peak at 12.5 min must be a higher molecular weight compound. The peak around 9.5 min is normally a trichlorobiphenyl while the peak around 7.0 min may be a dichlorobiphenyl. Previous reports showed the formation of hydroxylated-DCB, methylated-DCB and mono-chlorobiphenyl for the degradation of 2,6-DCB [51,52]. Another report confirmed that 2,6-DCB photodegradation yielded 2-chlorobiphenyl, 6-chlorobiphenyl and biphenyl [26]. Similar products may have formed in our study (Figure 1**)**. As seen in Figure 4 for 3,4′,5-TCB**,** some of the photoproducts are due to dechlorination to less substituted biphenyls [26,53–55].

### 3.3. Degradation Rate Constants and Half-Lives

The degradation of the 5 selected PCBs followed a pseudo-first order reaction in general [56,57]. The best linear fit yielded R^2^ > 0.92. However, for TiO_2_ photocatalyzed degradation, the linear fit was not optimal due to adsorption of PCBs onto the catalyst surface. The degradation kinetics involving H_2_O_2_ showed very good first-order fits. Their pseudo-first order degradation rate and half-lives were determined and listed in Table 2. Literature values for degradation half-lives in hexane irradiated by 254 nm UV lamp for some of the compounds in the present study are 2,6-DCB, 23 min; 2,2′,4,4′-TCB, 925 min; 3,3′,4,4′-TCB, >1,000 min. 2,2′,4,4′-TCB was efficiently degraded under all conditions, but under the condition with light + TiO_2_ + H_2_O_2_ being the fastest. The slow degrading compounds are 2,6-DCB, 3,4′5-TCB and 3,3′4′4′-TCB in both neutral and alkaline conditions. Peroxide can efficiently catalyze complete mineralization of most PCBs.

**Figure 5:**
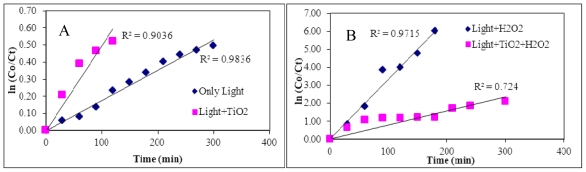
Degradation kinetics of 3,3′,4,4′-TCB (A) at pH 7 (B) peroxide catalysis.

A representative graph for the degradation kinetics with direct photolysis, TiO_2_ photocatalysis and peroxide catalysis is presented in Figure 5. 3,3′,4,4′-TCB which has all the 4 ortho positions vacant was highly resistant to photodegradation with very large half-lives (Figure 5A). Similar results were reported by Miao *et al.* [24] when the degradation was performed in UV light with hexane as solvent. However, peroxide as catalyst has greatly accelerated the degradation rate of 3,3′,4,4′-TCB with a t_1/2_ of 20 min and rate constant of 0.034 min^−1^. The efficiency of hydrogen peroxide in achieving faster degradation is due to the production of larger number of hydroxyl radicals [58,59]. Complete mineralization of 3,3′,4,4′-TCB could be achieved in a shorter time in the presence of hydrogen peroxide suggesting the success of this methodology.

### 3.4. Effect of PCB Structure on Degradation Rate

It can be clearly seen in Table 2 that the two planar PCBs, 3,4′,5-TCB and 3,3′,4,4′-TCB, degrade very slowly with degradation half lives of >4 hours. While the non-planar 2,6-DCB has a half-life around 2 hours and the perpendicular 2,3,5,6-TCB and 2,2′,4,4′-TCB degrades even faster with half lives of 107 and 38 min respectively. 2,3,5,6-TCB, and 2,2′,4,4′-TCB degrade very fast at pH 9 with estimated half lives of less than 10 min. Lin *et al.* reported that the number and location of chlorine atoms are important for PCB degradation [60]. Hong *et al.* [26] showed that photodegradation of 2,2′,4,4′-TCB in n-hexane with t_1/2_ of 926 min. In contrast, its degradation half life is <66 min in our study in the aqueous solvent. The difficulty in degrading planar PCBs like 3,3′,4,4′-TCB is due to the extended conjugation between the two phenyl rings leading to a longer half-life as reported by Miao *et al.* [24] It has been widely reported that ortho chlorine confers greater photolability on a PCB [27,61]. Similar results were observed for 3,3′,4,4′-, 3,3′,4,4′,5-, and 3,3′,4,4′,5,5′-PCBs by de Felip *et al.* [51]. All these three PCBs showed t_1/2_ values of >300 min which is in good agreement with our results. Our results further confirm that photoreactivity of PCBs depends on the position of the chlorines: ortho > meta > para positions of PCB rings [20].

### 3.5. Effect of TiO_2_, H_2_O_2_, and pH on the Photodegradation

The presence of H_2_O_2_ has a profound effect on photodegradation of non-planar PCBs which degrade generally very slow. The degradation rate enhancement is 20 times for 3,3′,4,4′-TCB and 35 times for 3,4′,5′-TCB. This great enhancement could be utilized as a possible “green” method for environmental remediation of planar PCBs. TiO_2_ alone does not have a strong effect on the photodegradation in aqueous solution. However, the combination of H_2_O_2_ and TiO_2_ speeds up the degradation for all the five tested PCBs. H_2_O_2_-catalyzed photodegradation of all 5 PCBs showed photoproducts of lower substituted biphenyls. The degradation was relatively fast (<30 min) for 2,2′,4,4′-TCB and 3,3′,4,4′-TCB without intermediate photoproducts detected. It was reported that irradiation of H_2_O_2_ with λ < 360 nm light leads to efficient generation of OH^•^ radicals [54]. The OH^•^ can dechlorinate PCBs and produce lower molecular weight products.

The use of light/H_2_O_2_/TiO_2_ for the degradation of many hazardous compounds has been reported in recent years. However, PCBs degradation using hydrogen peroxide as a catalyst was not reported to our knowledge [57,62–64]. H_2_O_2_ can absorb light energy from UV irradiation and the O-O bond is ruptured leading to the production of activated ^•^OH which is the crucial step in improving the rate of the reaction [62]. Other mechanisms proposed by Barkat *et al.* [65] suggested that H_2_O_2_ is a better electron acceptor than oxygen decreasing the chances of an electron/holes recombination [65]. The hydroxyl radicals formed by the photolysis of H_2_O_2_ might react with the holes generated during excitation which in turn will reduce the probability to undergo electron-hole recombination. The superoxide radical (^•^O_2_ ^−^) formed as the result is less dominant in affecting the rate of the photocatalytic reaction as compared with hydroxyl radicals. The general mechanisms of radical formation in various photocatalytic reactions are presented below.

$$\begin{array}{l} \mathrm{UV}/\mathrm{Ti}O_{2}\\ {\text{TiO}}_{2}+hv\to {\text{TiO}}_{2}({\text{e}}^{-}+{\text{h}}^{+})\\ {\text{TiO}}_{2}{\text{h}}^{+}+{{\text{OH}}^{-}}_{\text{ad}}\to {\text{TiO}}_{2}+{\text{OH}}^{•}\\ \mathrm{UV}/H_{2}O_{2}\\ {\text{H}}_{2}{\text{O}}_{2}+hv\to 2{\text{OH}}^{•}\\ \mathrm{UV}/\mathrm{Ti}O_{2}/H_{2}O_{2}\\ {\text{TiO}}_{2}+hv\to {\text{TiO}}_{2}({\text{e}}^{-}+{\text{h}}^{+})\\ {\text{TiO}}_{2}{\text{h}}^{+}+{{\text{OH}}^{-}}_{\text{ad}}\to {\text{TiO}}_{2}+{\text{OH}}^{•}\\ {\text{H}}_{2}{\text{O}}_{2}+{\text{e}}^{-}\to {\text{OH}}^{•}+{\text{OH}}^{-} \end{array}$$

In the present study the application of peroxide in the degradation of selected PCBs was found to be the most effective methodology. Degradation of 2,6-DCB in H_2_O_2_/UV and H_2_O_2_/UV/TiO_2_ showed 68% and 83% respectively. While 100% degradation of 2,3,5,6-TCB was seen using both methods. TiO_2_ presence has aided the degradation to completion in 60 min which otherwise took 90 min. For degradation of 2,2′,4,4′-TCB, UV/H_2_O_2_/TiO_2_ facilitated 100% degradation after 210 min of irradiation whereas 98% degradation was achieved in 270 min in the absence of the semiconductor catalyst. The effect of peroxide on coplanar PCBs, 3,4′,5-TCB and 3,3′4,4′-TCB, was slightly different from the previously discussed PCBs. Both these PCBs were highly resistant to degradation at pH 7 and partially degraded at pH 9. When peroxide was used in the presence of light, 100% degradation of the compound was achieved after 60 min of irradiation with no intermediates seen, while using H_2_O_2_/UV/TiO_2_ yielded 76% reduction at the end of 150 min.

## 4. Conclusions

Photodegradation of selected PCBs in CH_3_CN/H_2_O (80:20) medium is studied using simulated sunlight in the presence of TiO_2_ and H_2_O_2_ as catalysts. The overall photodegradation efficiency can be attributed to the fact that sunlight photocatalysis is more efficient for semi-conductor photocatalysis. Also, the solvent mixture CH_3_CN:H_2_O mimics real field condition. The photodegradation rate is dependent upon PCB structure and the use of TiO_2_ and H_2_O_2_. Quantum chemical calculations using density functional theory reveal that PCBs without any chlorine substitution on the *ortho*-position are closer to be planar, but PCBs with at least one chlorine substitution at the *ortho*-position causes the two benzene rings to be more perpendicular. The near planar PCBs degrade much slower than the near perpendicular PCBs. The use of nano-TiO_2_ speeds up the degradation of the planar PCBs, but has little effect on the degradation of the non-planar ones. The use of H_2_O_2_ greatly speeds up the degradation of planar PCBs as well as the non-planar 2,3,5,6-TCB, but has little effect on the other two non-planar PCBs. The relative degradation rate is: 2,2′,4,4′-TCB > 2,3,5,6-TCB > 2,6-DCB ≈ 3,3′,4,4′-TCB > 3,4′,5-TCB. The great enhancement of photodegradation rate by the addition of H_2_O_2_ may be further studied and used as a “green” methodology for some PCB remediation since the product of H_2_O_2_ is water and sunlight is natural and abundant.

## Figures and Tables

**Table 1:** Computational calculated structural properties of selected PCBs.

Name and Abbreviation	3-D Structure	Dihedral Angle	Position of Cl
3,3′,4,4′-Tetrachlorobiphenyl (3,3′,4,4′-TCB)	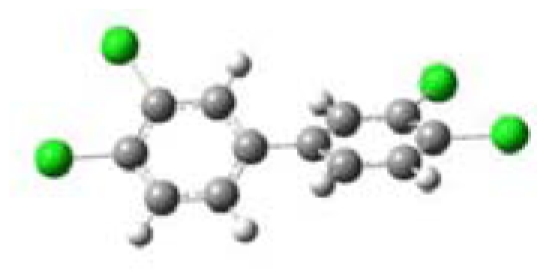	37.09	m,m′,p,p′
3,4′,5-Trichlorobiphenyl (3,4′,5-TCB)	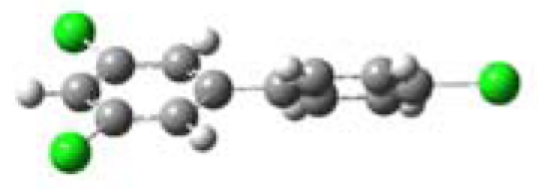	37.35	m,p′,m
2,2′,4,4′-Tetrachlorobiphenyl (2,2′,4,4′-TCB)	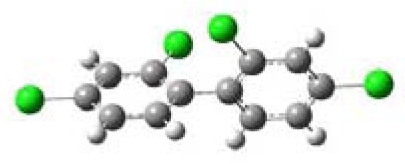	68.27	o,o′,p,p′
2,6-Dichlorobipheny (2,6-DCB)	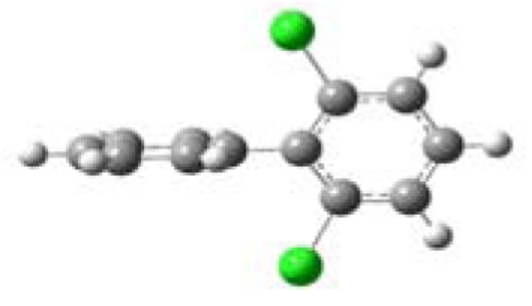	76.84	o,o
2,3,5,6-Tetrachlorobiphenyl (2,3,5,6-TCB)	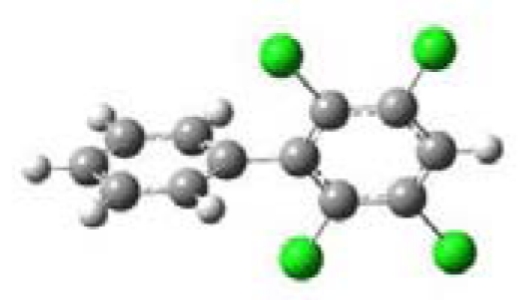	90.00	o,m,m,o

**Table 2:** PCB photodegradation half-life (t_1/2_) and first order rate constant (k, min^−1^).

PCBs	pH 7	pH 7+TiO_2_	pH 9	pH 9+TiO_2_	H_2_O_2_	H_2_O_2_+TiO_2_
2,6-DCB	139 min 0.0050	192 min 0.0036	157 min 0.0044	*b*	178 min 0.0039	110 min 0.0063
3,4′,5-TCB	630 min 0.0011	141 min 0.0049	533 min 0.0013	124 min 0.0056	18 min 0.038	55 min 0.013
2,3,5,6-TCB	107 min 0.0065	239 min 0.0029	*a*	*a*	48 min 0.0143	34 min 0.020
2,2′,4,4′-TCB	38 min 0.018	66 min 0.010	*a*	*a*	46 min 0.015	35 min 0.021
3,3′,4,4′-TCB	385 min 0.0018	161 min 0.0043	217 min 0.0032	128 min 0.0054	20 min 0.033	88 min 0.0079

a Experiments were not carried out since the degradation was very fast.

b Values were not calculated since degradation was <10%.
